# Student-Led Curricular Development in the Biomedical Science Master’s Program Using Virtual Dissection

**DOI:** 10.1007/s40670-023-01935-4

**Published:** 2023-11-16

**Authors:** Emre Coskun, Molly K. Beier, Kelsie N. Jackson, David R. Wang, Allison Seacat, Erica L. Ausel

**Affiliations:** College of Osteopathic Medicine, Marian University, 3200 Cold Spring Rd, Indianapolis, IN USA

**Keywords:** Anatomage, Educational technology, Curricular development, Future of academic medicine

## Abstract

The Anatomage table is a virtual dissection technology increasingly used to supplement anatomy education while its efficacy and integration is still being evaluated. To address the gap in student curricular leadership in such technological integration, previous master’s and current medical students led a curricular development effort to design, create, and incorporate hands-on Anatomage learning activities into a master’s level anatomy course during the 2021–2022 academic year. To assess changing perspectives regarding the table’s role in curriculum integration and content retention, surveys were conducted before and after voluntary participants completed the learning activities. Overall, participants had a more positive perspective on the integration of the Anatomage table into the curriculum and its use to retain material compared to non-participants. Participants were significantly more likely to feel motivated to learn anatomy while interacting with the table. Compared to peers who only experienced the table in didactic lecture, activity participants were significantly more likely to perceive that the table helped them learn the skeletal system, a perception supported by significantly higher scores on skeletal anatomy exam questions. Less positive perspectives were observed overall for the muscular system, demonstrating the efficacy of the table varied with content. This research contributes to our understanding of virtual technology in anatomy education, and, although the integration of student-developed activities was complex, various educational features and pedagogical approaches were successfully utilized to establish a novel supplemental resource that contributes to multimodal learning and an academic foundation that prepares learners for their future careers in biomedical sciences and medicine.

## Introduction

The Anatomage table (Anatomage Inc., San Jose, CA) is a virtual dissection technology that is becoming increasingly used to supplement the learning of anatomy in health sciences education. This tool is multifaceted with several advantages such as unlimited reusability, life-size and high-resolution cadavers with accurate anatomy, and educational features that can supplement foundational knowledge. In addition to supplementing anatomical education, the Anatomage table is also an example of growing access to technology in medical and graduate education, the efficacy of which has not been fully explored [1].

Virtual dissection technologies have been studied in various fields of education including undergraduate medical education, radiography, and surgical education [2–4]; however, research on the incorporation of the Anatomage into curricula is relatively limited. As virtual dissector tools become increasingly utilized in anatomy education, most research has focused on their perceived impact and outcomes. These studies demonstrate a general consensus among users that integration of the Anatomage table results in a positive impact on students’ motivation, satisfaction, engagement, and perceived effectiveness and learning [1–3, 5]. Importantly, these outcomes are factors that play an important role in retention of material by the learner. As virtual dissectors expand into the curriculum, research comparing different options among available technologies is also limited. This gap in the literature was explored by Vasil’ev and colleagues (2003) who compared the Anatomage table to a different virtual tool, the Pirogov interactive anatomy table. They found satisfaction was similar between faculty and students across multiple medical schools, but that each performed differently among various learner populations [6]. These studies demonstrate the potential of the Anatomage table in anatomy education, but, to the best of our knowledge, no study has used learner perspectives to pursue a student-led curricular design to evaluate the incorporation of the Anatomage table into an existing anatomy curriculum.

By incorporating these types of technological advancements into medical education, this tool can also address concerns related to cadaveric dissection. Cadaveric dissection has been considered the gold-standard tool for incorporating hands-on experience into anatomy education and achieving greater insight into the human body for generations of learners [7, 8]. However, the use of cadavers presents constraints related to costs, shortages of cadavers, the inefficient use of time spent identifying difficult structures, reduction in curriculum time for dissection, labor intensiveness for anatomy faculty, and the inability to reuse cadavers [9]. These concerns have encouraged the use of virtual dissection technology such as that found on the Anatomage table. Ultimately, any single teaching modality such as didactic teaching, models, cadaveric dissection, atlases, computer-based, or case-based learning has not been shown to fulfill all aspects of medical curriculum, and a multimodal approach to learning anatomy is still considered ideal [10, 11]. Therefore, the Anatomage table could be a valuable supplemental resource for learning anatomy due to its educational features, especially if cadaveric dissection is difficult to establish or unavailable.

### Project Goals

The Biomedical Sciences Master’s (BMS) program at Marian University’s College of Osteopathic Medicine (MU-COM) aims to prepare students for the next steps of their education and careers in medicine and biomedical sciences. One aspect of the program is the comprehensive curriculum including a two-semester BMS 521/522 Anatomy, Histology, and Embryology (AHE) course set. The instructional material for this course consists of lectures and two-dimensional illustrations from textbooks, atlases, and videos, but is unable to include cadaveric dissection due to constraints including limited time and credit hours.

Current MU-COM medical students and graduates of the BMS program led a curricular development effort to address the gap in student curricular leadership in virtual dissection advancements and to support a multimodal approach to learning anatomy in the MU-COM BMS program. As their BMS capstone projects during the summer of 2021, they designed and created hands-on learning activities using the Anatomage table to directly supplement the lecture content in the BMS anatomy course. The goal of these activities was to provide a novel, sustainable, and interactive resource for 3-D visualization of course material for future learners. The following academic year, as medical students at MU-COM, they planned and tested the integration of those activities into the BMS anatomy course, collected student feedback, and analyzed perceived learning outcomes.

## Materials and Methods

### Curriculum Design and Related Pedagogy

The Backward Design Process was primarily used to design and create the learning activities. This is a specific method of organization used to attain adequate alignment between course objectives, lecture content, and assessments in the curriculum [12]. It begins with the course vision which gives rise to the Course Learning Objectives (CLOs). The existing CLOs for the BMS anatomy course were used as broad learning outcomes to guide the creation of new and unique module-level learning objectives for each supplemental activity created on the Anatomage table. The rest of the Backward Design concepts, such as the deliverables and content, were incorporated throughout the activities to ensure each was appropriate for the intended audience. Finally, formative post-activity assessments were created for every activity which were linked to the learning objectives and provided an opportunity for students to assess their understanding of the material. Each student researcher was independently responsible for this process for each Anatomage activity they created.

When creating the unique learning objectives for the Anatomage activities, action verbs were selected based on Bloom’s Taxonomy [13]. Bloom’s Taxonomy categorizes action verbs to emphasize how the material should be studied and tested. Incorporating Bloom’s Taxonomy into the Backward Design Process was an important point in the curriculum design effort to gauge the proposed activity outcomes and a valuable opportunity to apply the students’ research and understanding of curriculum design. Wide range of action verbs across various levels of learning were utilized when writing the activities’ learning objectives. This provided an opportunity to consume the content in multiple ways, align activity learning objectives with the CLOs, and to integrate various capabilities of the Anatomage table at the same time. This was time-consuming, but the outcome was rewarding because it ensured that the activities were useful and purposely aligned with the course content.

### Student Curricular Leadership

The student researchers were preparing to begin medical school at the time activities were created, and they desired to use their knowledge of the cadaver lab to apply key concepts and known benefits of cadaveric dissection to the design of the Anatomage activities. Physical dissection is perceived among students to improve teamwork, professionalism, and understanding of structural relationships in human anatomy [14], particularly when it is conducted in a small group setting [15]. Such aspects of cadaveric dissection can be mirrored to ensure that the Anatomage table is an effective supplemental resource. For example, the Cardiovascular System module was designed to mirror cadaveric dissection in small groups by utilizing the high resolution and life-sized display to make precise incisions on the virtual cadaver and remove structures individually. Inspired by a traditional gross anatomy dissector manual offered to medical students at MU-COM [16], this specific activity guided students to dissect and explore the heart and its relationship to surrounding structures. This additionally ensured that the module covered the same high-yield material expected in medical school.

Student researchers’ previous experience in the BMS program and the AHE courses played a crucial role in recognizing anatomy content that would benefit the most from supplemental 3-D visualizations. Anatomage activities were designed based on topics that the researchers thought were most difficult to understand or visually conceptualize during didactic lectures to strengthen future learners’ comprehension of those topics. This process also included faculty oversight, which is discussed below. Once challenging content was identified, various educational tools available on the Anatomage table were explored by each researcher independently to find a suitable approach to use in their activity. Of note, the variety of features available in the Anatomage table was ideal for its consideration as an additional modality in BMS anatomy course. This variety allowed the researchers to supplement any organ system covered in the course using a variety of interactive methods. The features utilized to create the supplemental activities include virtual dissection, structure identification via recall, tracing of blood flow, histology, visualization of physiological pathways, and case studies with medical imaging.

The unique perspective of previous BMS students and their role as student researchers in curriculum design was also an opportunity for collaboration between faculty and students. Students do not often experience the developmental aspect of educational material, which proved to be informative in many ways. Both content selection and its delivery required consideration of several factors. These included an evaluation of the researchers’ own knowledge gaps in supplemented topics, recognition that innovative technologies come with learning curves, and the awareness of learner cognitive load. In addition, creating assessments to measure student progress motivated researchers to be intentional with their activities. The exposure to Anatomage cadavers also helped solidify those knowledge gaps and was a valuable opportunity to prepare for learning in the cadaver lab in the upcoming semester. Ultimately, this reflective and interactive approach provided understanding for the processes behind teaching in higher education, appreciation of the work that goes into developing an effective curriculum, and a perspective that will allow for effectively educating patients, their families, and medical students as future physicians.

### Faculty Oversight

The capstone projects described in this manuscript began in the summer of 2021 under the supervision of the final author listed (Ausel), the course director of BMS 521/522 AHE for which this project was created. Ausel had two goals in facilitating this project: (1) create novel course content using 3D technology utilizing the combined bandwidth of Master’s students and (2) incorporate student experiences and perceptions of the content to create high impact active learning activities for future learners.

Prior to the creation of the Anatomage activities, the students and Ausel met as a group to outline shared expectations and goals to create consistency between projects. The following was outlined: identify content in each anatomical system that student researchers/peers struggled to learn during the course; create an interactive learning activity on identified content using the Anatomage table to serve as a supplement to didactic lecture; employ AHE course learning objectives as a guide; and build Canvas modules for each activity following the same organization which should include course learning objectives, activity learning objectives, activity description and instructions, and a brief assessment for participants to complete following the activity. Each activity creator was also expected to explore relevant literature on pedagogical methods and curricular design to integrate into a final manuscript alongside a description of their activity with pros and cons of the creation process. It is Ausel’s opinion that this initial group meeting was essential for creating a resource that was approachable and consistent for future learners and to build this project as participant action research [17].

After this initial meeting, Ausel met individually with each student throughout the summer to provide Anatomage table training, discuss ideas, revise activity specific learning objectives, and review each activity. Common themes during these meetings included how to write learning objectives for students to achieve a specific goal and creating an activity that focuses on the strengths and unique features of the Anatomage table. For the creators’ final assessment, Ausel completed each activity using the Canvas modules and provided feedback to incorporate before the pilot project for the upcoming semester began. Before the implementation of these activities into the course, Ausel also added images of the table’s buttons and menus to the instruction to ease the learning transition for Anatomage Table Pilot participants.

### Project Implementation

At the beginning of the Fall 2021 semester, students in AHE were introduced to the Anatomage table during lecture. Following this, the authors asked for volunteers to participate in the Anatomage table Pilot project. Because this supplementary material is viewed as beneficial, the authors requested volunteers rather than randomized participants to maintain fairness in the course. A total of 24 of 51 students chose to participate. Participants were added to a Canvas course (available here https://lor.instructure.com/resources/51a17ded397d4acfb666c468c558c825) where all student researcher created activities were housed. Canvas modules were made available after associated material was taught via didactic lecture. Four activities per semester were opened and students signed up in small groups of 4 to 5 to complete each in Marian University’s simulation lab which houses the Anatomage table. Ausel was present during the initial activity for each group to provide basic training and was accessible for in-person guidance during all others. Because each activity included detailed written instructions with each Canvas module, no other instructors were present during the activities. Pros and cons of this approach will be discussed below.

It is important to note here that all AHE students during the 2021–2022 academic year were exposed to the Anatomage table. The tool was used to project virtual cadavers on the normally employed lecture hall screens during various didactic lectures, specifically when structure orientation and spatial relationships were discussed. For this manuscript, in-class use of the Anatomage table featured the skeletal and muscular systems. However, it was only the pilot project participants who directly interacted with the Anatomage table.

### Survey Instrument

The authors aimed to measure changes in student perspective after experience with the table during the semester and between Anatomage Table Pilot participants and non-participants. To increase participation, two surveys were conducted during class-time distributed via a Qualtrics QR code. Students were informed that the surveys were IRB exempted (reference number S21.290), told the purpose of the survey, and that it was voluntary and had no bearing on their grade. Survey 1 was given at the beginning of the fall semester prior to the implementation of the Anatomage Table Pilot project. Survey 2 was given after the midterm exam, which assessed the musculoskeletal system, and followed in-class use of the table during lecture alongside two pilot activities.

Both surveys were built using a 5-point Likert scale (strongly agree (StA), somewhat agree (SoA), neither agree or disagree (N), somewhat disagree (SoD), strongly disagree (StD)), and asked students a series of statements that considered the integration of virtual technology/Anatomage table into anatomy curriculum. Survey 1 asked students (1) whether virtual technology should be included in anatomy curriculum; (2) whether virtual technology is superior to traditional teaching methods for anatomy; (3) whether they feel motivated to learn anatomy using virtual technology; and (4) whether they feel comfortable with using virtual technology. Survey 2 focused specifically on the Anatomage table and asked (1) whether the Anatomage table should be included in anatomy curriculum; (2) whether the Anatomage table is superior to traditional teaching methods for anatomy; (3) and whether students feel motivated to learn anatomy using the Anatomage table. In addition, survey 2 asked students about the role of the Anatomage table in their perceived retention of course material asking (1) whether the Anatomage table helped them learn the bones of the body; (2) whether the Anatomage table helped them learn the muscles of the body; (3) whether the Anatomage table helped them contextualize structures in the body; and (4) whether the Anatomage table helped them succeed on exams. The authors recognize the shift in wording between the two surveys, from “virtual technology” to “the Anatomage table,” however feel that it is acceptable as the Anatomage table was unknown to most students when survey 1 was implemented.

## Results

For survey 1, a total of 51 (100%) students participated. Survey 2 saw lower participation at 42 (84% as one student dropped from the course) students. The statistical software SPSS 29.0.1.0 was used to examine these data. Both Likert-scale surveys were tested for their reliability and validity. The result of Cronbach’s alpha analysis of survey 1 was 0.666, which falls slightly below the standard level of reliability at 0.70. In comparison, Cronbach’s alpha analysis for survey 2 was 0.903, demonstrating its reliability. Construct convergent validity for each survey was examined using a two-tailed Pearson’s correlation test; the total score of responses correlated significantly (*p* ≤ 0.002) with individual responses for all statements, demonstrating the surveys’ validity. Because all survey statements were written based on a centralized theme and expected to correlate, discriminant validity was not tested. It is recognized that these data reflect the validity and reliability of a novel survey, but will be used to guide future research using these assessments.

### Surveys 1 and 2: Perspective Variation After Anatomage Table Incorporation

**Fig. 1:**
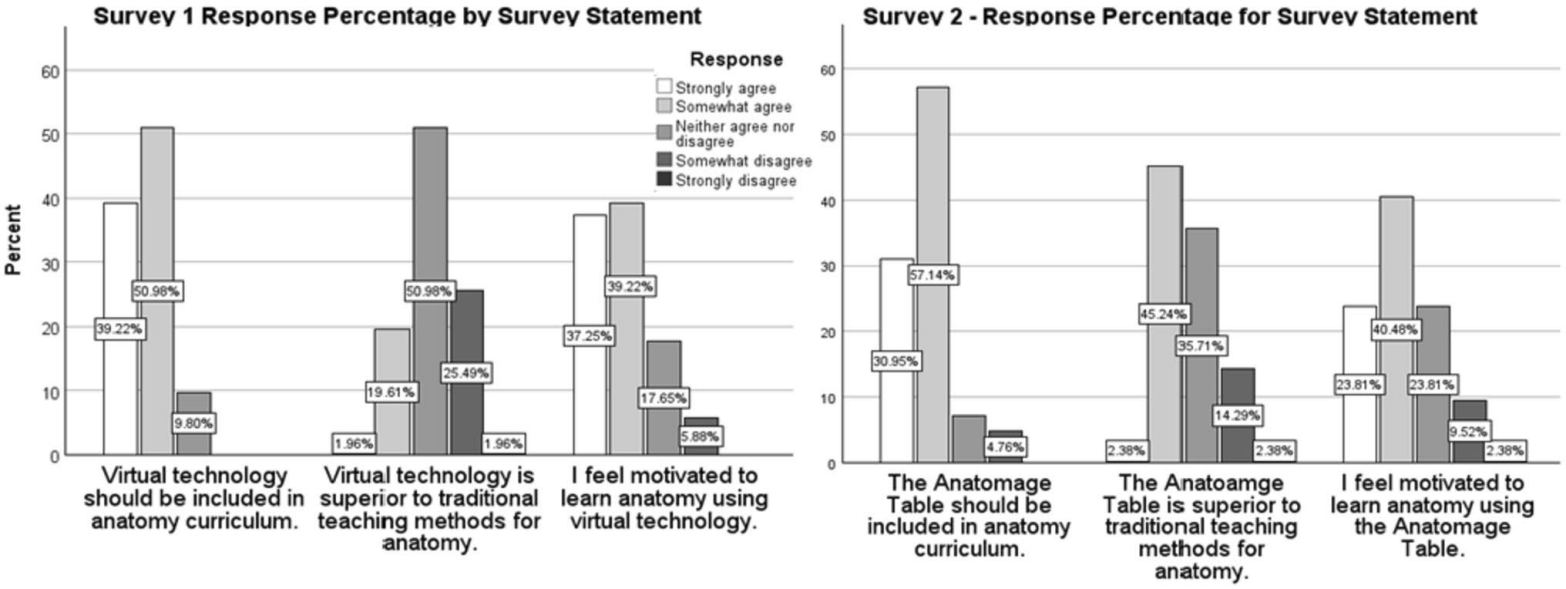
Response distribution for Survey 1 and Survey 2

The distribution of responses for surveys 1 and 2 were compared to assess changes in perspectives regarding the use of virtual technology after the implementation of the Anatomage Table Pilot project and its in-class implementation. Figure 1 displays the distribution of responses for each survey. A Mann-Whitney *U* independent *t* test was employed to compare the distribution of each statement asked on both surveys (Table 1). Among all survey responses, there was a slight decline among students who agreed the table should be included in the curriculum or were motivated by it to learn. In comparison, there was a significant shift among those who agreed with the statement that the technology is superior to traditional teaching methods. It is important to note that superiority applies to a comparison with existing resources in the BMS anatomy curriculum such as atlases, videos, and diagrams. It did not include cadaveric dissection as this is not a part of the curriculum, which was clarified during the administration of both surveys.

**Table 1:** Comparison of first and second survey response distribution of Anatomage perspective survey

Survey question	Survey	Number	Mean (SD)	Median	*U*-value
*1. Virtual technology/the Anatomage table should be included in anatomy curriculum*	1	51	1.86	2	1169.50
	2	42	1.71	2	
*2. Virtual technology/the Anatomage table is superior to traditional teaching methods for anatomy*	1	51	2.69	3	786.00*
	2	42	3.06	3	
*3. I feel motivated to learn anatomy using virtual technology/the Anatomage table*	1	51	2.26	2	12.72.00
	2	42	1.92	2	

*p = 0.019

The results for the first statement—*Virtual technology/The Anatomage table should be included in anatomy curriculum*—from survey 1 demonstrates that students primarily thought virtual technology should be included in anatomy curriculum (StA, 39.22%; SoA, 50.98%; N, 9.80%). No respondents selected either disagree statement in survey 1 (Fig. 1), compared to 4.76% of respondents who selected somewhat disagreed in survey 2. Between surveys 1 and 2, there was a shift in perspective away from those who strongly agreed that the table should be included in the curriculum. Respondents who strongly agreed shifted from 39.22% in survey 1 to 30.95% in survey 2 and those that somewhat agreed shifted from 50.98% in survey 1 to 57.14% in survey 2. This shift was not significant but suggests a more measured perspective of virtual technology after its implementation.

Figure 1 shows the distribution of responses for each of the three statements included on both surveys 1 and 2, represents changing student perspective before and after the implementation of the Anatomage Table Pilot Program and use of the technology in-class.

Opposing the results described above, responses shifted towards agreement for the second statement—*Virtual technology/The Anatomage table is superior to traditional teaching methods for anatomy*—between surveys 1 and 2 (Fig. 1). A decrease in the percentage of respondents who disagreed (survey 1, 27.45%; survey 2, 16.67%) and neither agreed nor disagreed (survey 1, 50.96%; survey 2, 35.71%) with the statement was observed between surveys 1 and 2. Meanwhile, the percentage of those who somewhat agreed that the Anatomage table is superior to traditional teaching methods increased from 19.61% in survey 1 to 45.24% in survey 2. Only minor changes were observed in the strongly agreed response (survey 1, 1.96%; survey 2, 2.38%). This change in distribution, driven by an upward trend among those who somewhat agreed with the statement, was significant (*p* = 0.019) (Table 1).

There was no significant difference between surveys 1 and 2 for the third survey statement—*I feel motivated to learn anatomy using virtual technology/the Anatomage table.* However, like the first statement, there was a downward shift among participants who strongly agreed that they were motivated by the technology (Table 1). In survey 1, 37.25% of students felt strongly motivated compared to 23.81% in survey 2. While the percentage of responses deviated little for those who somewhat agreed (survey 1, 39.22%; survey 2, 40.48%), those who neither agreed nor disagreed and those who somewhat disagreed with the statement rose from 17.65 to 23.81% and 5.88 to 9.52%, respectively. While neither the first nor the second statement in the survey demonstrated significant changes, both trended from agreement towards disagreement.

Table 1 shows the comparison of questions shared between survey 1 and survey 2 using a Mann-Whitney *U* test.

### Anatomage Table Pilot Project: Participants’ and Non-Participants’ Perspectives

#### Curriculum Integration Perspective

Perspectives among Anatomage Table Pilot participants and non-participants were compared using responses from survey 2 (Tables 2 and 3). A total of 20 participants and 22 non-participants responded to the second survey. Overall, pilot participants were more likely to strongly agree with the statements related to the incorporation of the table into anatomy curriculum (statements 1–3, Table 2) and less likely to neither agree nor disagree. While 90% of participants and 86.36% of non-participants agreed that the table should be included in anatomy curriculum, 45.00% of participants versus 18.18% of non-participants strongly agreed. While the difference in response distribution does not differ significantly (*p* = 0.091), it corresponds to other trends observed between these two groups.

**Table 2:** Second survey results by pilot participants and non-participants, subdivided by perspective on curriculum integration and content retention

Survey question	Anatomage pilot participants (N)	Strongly agree	Somewhat agree	Neither agree nor disagree	Somewhat disagree	Strongly disagree
Perspective on curriculum integration						
*1. The Anatomage table should be included in anatomy curriculum*	Participant (20)	45.00%	45.00%	10.00%	0.00%	0.00%
	Non-participant (22)	18.18%	68.18%	4.55%	9.09%	0.00%
*2. The Anatomage table is superior to traditional teaching methods for anatomy*	Participant (20)	5.00%	50.00%	30.00%	15.00%	0.00%
	Non-participant (22)	0.00%	40.91%	40.91%	13.64%	4.55%
*3. I feel motivated to learn anatomy using the Anatomage table*	Participant (20)	40.00%	40.00%	10.00%	5.00%	5.00%
	Non-participant (22)	9.09%	40.91%	36.36%	13.64%	0.00%
Perspective on content retention						
*4. The Anatomage table helped me learn the bones of the body*	Participant (20)	55.00%	30.00%	15.00%	0.00%	0.00%
	Non-participant (22)	27.27%	36.36%	22.73%	9.09%	4.55%
*5. The Anatomage table helped me learn the muscles of the body*	Participant (20)	15.00%	55.00%	30.00%	0.00%	0.00%
	Non-participant (22)	13.64%	45.40%	27.20%	4.55%	9.09%
*6. The Anatomage table helped me contextualize structures in the body*	Participant (20)	60.00%	30.00%	10.00%	0.00%	0.00%
	Non-participant (22)	45.45%	50.00%	4.55%	0.00%	0.00%
*7. The Anatomage table helped me succeed on exams*	Participant (20)	25.00%	45.00%	20.00%	10.0%	0.00%
	Non-participant (22)	13.64%	36.36%	40.91%	0.00%	9.09%

**Table 3:** Comparison of second survey response distribution between Anatomage Table Pilot Program participants and non-participants

Survey question	Subgroup (*N*)	Mean (SD)	Median	*U*-value
Curriculum perspective				
*The Anatomage table should be included in anatomy curriculum*	Pilot participant (20)	1.65 (0.671)	2	279.5
	Non-participant (22)	2.05 (0.785)	2	
*The Anatomage table is superior to traditional teaching methods for anatomy*	Pilot participant (20)	2.55 (0.826)	2	255.5
	Non-participant (22)	2.82 (0.853)	3	
*I feel motivated to learn anatomy using the Anatomage table*	Pilot participant (20)	1.95 (1.09)	2	307.5*
	Non-participant (22)	2.55 (0.858)	2.5	
Content retention perspective				
*The Anatomage table helped me learn the bones of the body*	Pilot participant (20)	1.60 (0.754)	1	297.5**
	Non-participant (22)	2.27 (1.120)	2	
*The Anatomage table helped me learn the muscles of the body*	Pilot participant (20)	2.15 (0.671)	2	251.5
	Non-participant (22)	2.50 (1.102)	2	
*The Anatomage table helped me contextualize structures in the body*	Pilot participant (20)	1.50 (0.688)	1	244.0
	Non-participant (22)	1.59 (0.590)	2	
*The Anatomage table helped me succeed on exams*	Pilot participant (20)	2.15 (0.933)	2	267.5
	Non-participant (22)	2.55 (1.057)	2.5	

*p = 0.021; **p = 0.039

Table 2 shows the results of the second survey by pilot participants and non-participants and is subdivided by perspective on curriculum integration and perspective on content retention by the respondents.

Table 3 shows the comparison of pilot participant and non-participant response distribution for survey 2 using a Mann-Whitney *U* test.

**Fig. 2:**
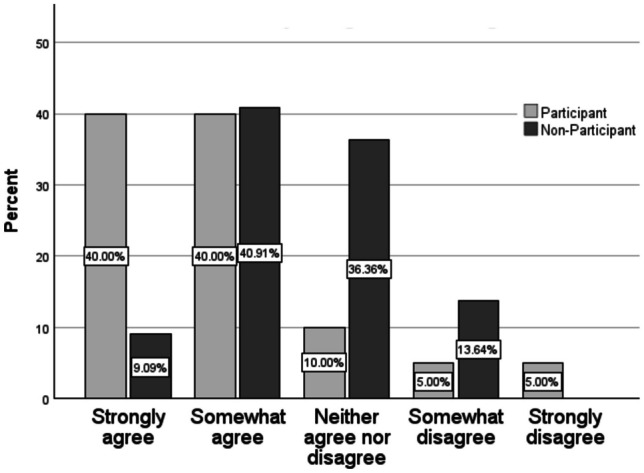
Response distribution for anatomage table pilot project participants and non-participants for the statement- I feel motivated to learn anatomy using the anatomage table

A similar pattern was observed for participants and non-participants when asked about their motivation to learn anatomy by using table (Table 2; Fig. 2). Among participants 40.00% strongly agreed and 40.00% somewhat agreed that the virtual tool motivated them. In comparison 9.09% and 40.91% of non-participants responded to the same statement with strongly or somewhat agreed, respectively. Moreover, only 10.00% of participants responded that they neither agreed nor disagreed with the statement, compared to 36.36% among non-participants. A minority of students did not feel motivated to learn anatomy using the Anatomage table (participants: SoD 5.00%, StD 5.00%; non-participants; SoD 13.64%, StD 0.00%). The distribution of these differing perspectives on motivation were significantly different from one another (*p* = 0.021) (Table 3).

Responses between participants and non-participants varied only slightly for the statements that the Anatomage table is superior compared to traditional teaching methods (Table 3). More pilot participants indicated that they agreed with the statement (StA, 5.00%; SoA, 50.00%) compared to non-participants (StA, 0.00%; SoA, 40.91%), and fewer participants neither agreed nor disagreed (participants, 30.00%; non-participants, 40.91%). The distribution of responses to this statement did not differ significantly.

Figure 2 shows the distribution of responses to the statement “I feel motivated to learn anatomy using the Anatomage table” among pilot participants and non-participants, a distribution that is significantly different between the two groups.

#### Content Retention Perspective

Four statements were included in survey 2 that focused on the perceived retention of material due to the integration of the Anatomage table. Overall, those who participated in the Anatomage Table Pilot were more likely to agree with the retention statements compared to non-participants (Table 2). In fact, only one statement—*The Anatomage table helped me succeed on exams*—showed any disagreement (SoD, 10.00%) among participants, a pattern not observed among non-participants.

**Fig. 3:**
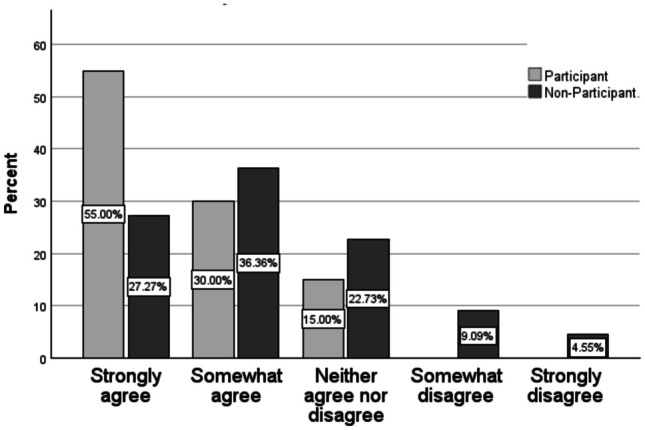
Percentage of responses for anatomage table pilot project participants and non-participants for survey question: The anatomage table helped me learn the bones of the body

Two of the statements focused on retention of course material, specifically the bones and muscles of the body. The distribution of responses varied significantly between participants and non-participants for the skeletal system (*p* = *0.039*) but not for the muscular system (Table 3). Among participants, 55.00% strongly agreed, 30.00% somewhat agreed, 15.00% neither agreed nor disagreed, and none disagreed with the statement that the table aided them in learning the bones of the body (Fig. 3; Table 2). In comparison, non-participants had a wider range of responses with less in agreement with the statement. Among non-participants, 27.27% strongly agreed, 36.36% somewhat agreed, 22.73% neither agreed nor disagreed, 9.09% somewhat disagreed, and 4.55% strongly disagreed that the Anatomage table helped them learn the bones of the body. In comparison, the distribution of responses to *The Anatomage table helped me learn the muscles of the body* among participants and non-participants were similar (Table 2), outside of 13.64% of non-participants and 0.00% of participants disagreeing with the statement.

Figure 3 shows the distribution of responses to the statement “The Anatomage table helped me learn the bones of the body” among pilot participants and non-participants, a distribution that is significantly different between the two groups.

A notable pattern emerged for the statement *The Anatomage table helped me contextualize structures in the body*. While more pilot participants strongly agreed with the statement (60.00%) compared to non-participants (45.45%), no students who partook in the survey disagreed that using the table helped them contextualize anatomical structures.

For the final statement—*The Anatomage Table helped me succeed on exams*—25.00% and 45.00% of participants strongly and somewhat agreed with the statement compared to 13.64% and 36.36% of non-participants (Table 2). The percentage of respondents who neither agreed nor disagreed with the statement was twice as high among non-participants at 40.91% compared to 20.00% in participants. Both groups had some respondents disagree with the statement; 10.0% of participants somewhat disagreed, and 9.09% of non-participants strongly disagreed.

## Discussion

The student curriculum leadership perspective and the addition of learning activities that provide direct hands-on interaction with the Anatomage table offers an informative view of novel technology in medical education. The survey results reflect similar positive outcomes reported by previous researchers that evaluated this technology. One 2013 study showed that when using technology, medical students were more engaged and more independent with lower self-reported reliance on other resources during dissection [18]. Another study demonstrated that technology positively impacted medical students’ perception of success and increased their excitement for the course material [1]. Here, activity participants had a more positive perspective on the integration of the Anatomage table into the anatomy curriculum, its use to retain material and as a motivation to learn anatomy. In addition, they were less likely to be undecided, to disagree with the utility of the table, and more likely to strongly agree with the survey statements when compared to non-participants. This likely stems from their direct interaction with the technology. Since the majority of BMS students continue their education and careers within health sciences and medicine, the participants’ motivation to use the table was rewarding to see due to the long-term impact this may have on their future education.

Despite overall positive perception the participants had towards the Anatomage table, there was a slight decline among those who strongly agreed with virtual technology being included in the anatomy curriculum and an increase among those who thought it should not. This may be due to the approach of in-class integration. Themes among open responses were that it was difficult to translate the anatomical images from lecture slides to the Anatomage cadavers when projected in the lecture hall combined with the shift in perspective to observing a prone horizontal cadaver. These aspects provide insight from the learner perspective that can be corrected in the future.

A significant shift in perspective regarding the superiority of the Anatomage table compared to traditional teaching methods occurred after the technology was introduced to students. It is important to note again that superiority refers to a comparison with existing resources in the BMS anatomy curriculum, and not cadaveric dissection. The comparison of pilot participants and non-participants in survey 2, which showed little variation between the two groups, demonstrates that this perspective was supported by those who did and did not experience hands-on interaction with the table. In sum, this change in perspective on the superiority of the device was driven by the mere presence of the Anatomage table and possibly its immense capabilities. A shared shift in perspective in both participant groups, compared to survey 1, was also observed when students responded to the statement that the table contextualized anatomical structures. None of the respondents, activity participants or not, disagreed with this statement in the second survey. These results, taken together, suggest that students overall appreciated the utility of this technology but integration into the classroom setting was difficult. Strong perspectives on the integration of technology into the course were tempered after its use; this highlights that how the table is integrated is important. In-person hands-on activities were perceived more positively by students compared to lecture context. But, this project has helped identify aspects to improve, such as helping students orient to horizontal prone cadaver views for use in lecture and more limitations discussed below.

An interesting pattern was observed when students were asked about retention of material based on the use of the Anatomage table. Pilot participants were significantly more likely to agree with the statement that the table helped them succeed on exams and aided in learning the skeletal system. To examine if their perspective related to their actual retention, the result of exam questions based on identification of gross skeletal structures were compared between participants and non-participants; participants achieved a significantly higher score on these questions compared to non-participants (92.86% vs. 85.45; *p* = 0.012; Table 4). The same approach was used to examine questions on skeletal muscle identification exam questions; pilot participants performed similarly to (88.28% vs. 86.11%; *p* = 0.447) their non-participant counterparts, reflecting the responses from survey 2 that indicated the two groups had a similar perspective on if the table helped them learn the muscle of the body. In sum, these data suggest that (1) the Anatomage table activity created for the skeletal system improved the perception and retention of knowledge and (2) the activity created, or the capabilities of the Anatomage table, did not have the same effect for the muscular system content. Ultimately, what material is taught on the Anatomage table matters; the perspective of students, activity type, and instruction, influence what type of material is retained.

**Table 4:** The percentage correct of exam questions related to gross skeletal and gross muscular anatomy between pilot participants and non-participants

		Skeletal identification		Muscle identification	
Anatomage Table Pilot	*N*	Mean (SD)	*p*	Mean (SD)	*p*
Participant	24	92.86 (8.15)	*0.012*	88.28 (10.4)	0.447
Non-participant	27	85.45 (11.96)		86.11 (11.1)	

Table 4 presents the percentage correct of exam questions related to gross skeletal anatomy and gross muscular anatomy between pilot participants and non-participants.

### Implementation Limitations

While our analysis provides additional insight into the benefits of utilizing the Anatomage table, there were some challenges to how it was integrated into the curriculum. One that relates to technological advancements in anatomy education was students learning how to use the table effectively. As with all technology, there is a learning-curve when first using a new device. It takes time to understand what each icon indicates, all the functions the table has, and how to be efficient with those features. There was also a period of time between each activity where students did not use the table, requiring them to re-learn some of these features. Based on our experience with designing and creating the activities, it is reasonable to expect that there could also be issues with excessive loading time, poor response time to desired actions, and freezing of the program. The level of comfort regarding technology also varies between students, with some students enjoying technology-based learning and others preferring more traditional resources. Overall, technological challenges may have also contributed to slight downward trends in curriculum integration perspectives.

The data collection method using the survey instrument also presented some challenges. A disadvantage to the survey instrument was the small sample size, which should be kept in mind while interpreting the results. Also, while each student who completed the surveys chose a unique identifier number that allowed us to track changes in perspective between the two surveys, some did not include their identifiers in both of their responses. The lack of consistency in students using their unique identifier number made it more difficult to follow trends of responses. In addition, not all students completed both surveys. This decreased our sample size and caused a discrepancy between the number of students that participated in the learning activities (24) and the number of participants that completed the survey (20). There was a similar discrepancy for non-participants.

Furthermore, participation in the learning activities was also voluntary and some participants failed to complete all modules, especially towards the end of the semester after survey 2 was administered. Similar to technological challenges, other causes of potential frustration or loss of interest could be burnout towards the end of the semester or the location and accessibility of the table. The table was available during common working hours, 8:00 am to 4:30 pm Monday through Friday at the simulation lab, but many students complete their work on campus outside of these hours. A graded activity would likely have achieved more consistency in activity completion and survey participation.

The variety of learning styles and approach to course material could also be a barrier to motivation and excitement to utilize the Anatomage table. Although having student-created activities allows for a unique perspective on what material is challenging or can benefit the most from supplementation with an additional resource, each student’s perspective is different and may not apply to all future learners. This may have resulted in learners spending extra time on material they did not deem overly challenging or in need of supplementation with a 3-D or interactive resource.

Beyond the limitations of using novel technology and its integration into our pilot study, we also reflected on the pedagogical pieces utilized. We created our learning activities based on various levels of learning described by Bloom’s Taxonomy which is used throughout other aspects of the MU-COM curriculum. However, an observation that the deeper and more comprehensive aspects of learning were challenged when using the Anatomage table due to lack of haptic feedback and tactile representation was noted. Haptic feedback has been demonstrated to be important to the deeper aspects of learning anatomy, specifically recall of anatomical structures and spatial orientation [19]. As a result, we could only expect proficiency in the lower levels of Bloom’s Taxonomy. This reflection was only possible from our own cadaver laboratory experiences a few short months into the medical school curriculum when we could appreciate the advantages of physical gross dissection. Specifically, the textures of organs were represented only by a typical touch screen, whereas it was a significant part of understanding different physical characteristics of tissues in the cadaver lab. This is a noteworthy reflection for the application of Anatomage, as the tactile representations that come with cadaveric dissection have been thought to promote a deeper understanding of spatial relationships in anatomy [20]. Despite the lack of haptic feedback, we agreed that the Anatomage table still has significant potential to help students better understand visual relationships in anatomy, relative to 2D illustrations, due to life-size representations that can be manipulated.

### Next Steps

The diverse range of topics that our learning activities supplement will establish a foundation to explore how the Anatomage table impacts anatomy education. Current research shows the integration of technology in this environment is complex. To establish its efficacy, additional research is necessary to better understand how and if the Anatomage table impacts the long-term retention of human anatomy. Our Anatomage activities, which supplement each organ system covered in the course set, sets a template for such research. Our goals with the Anatomage table in the future will be to address the limitations of activity implementation, optimize the logistics and content of learning activities, and further its integration into the BMS anatomy curriculum. We are especially interested to see how a novel resource created by students-turned-educators might impact their learning outcomes for future students.

## Conclusion

The Anatomage table is an important technological advancement in biomedical science education. Our supplemental activities take full advantage of the capabilities of this virtual dissection technology to support students’ anatomy education, and, therefore, it will be valuable to continue to reflect upon their impact. Overall, we have achieved our goal of designing and creating learning activities using the Anatomage table to directly supplement lecture content in the BMS anatomy curriculum. The Anatomage activities also support the purpose of the BMS program at MU-COM by helping students develop a strong academic foundation to ultimately prepare them for their future careers in biomedical sciences and medicine. This resource and the interactive learning activities that contribute to a multimodal method of learning can be implemented for teaching anatomy in many undergraduate, graduate, or medical educational programs.

## Data Availability

The data that support the findings of this study are available from the corresponding author, E.C., upon reasonable request.
